# Patients with persistent medically unexplained symptoms in general practice: characteristics and quality of care

**DOI:** 10.1186/1471-2296-8-33

**Published:** 2007-05-31

**Authors:** Anja JE Dirkzwager, Peter FM Verhaak

**Affiliations:** 1 Netherlands Institute for Health Services Research (NIVEL), Utrecht, the Netherlands

## Abstract

**Background:**

Medically unexplained physical symptoms (MUPS) are common in general practice (GP), and are even more problematic as they become persistent. The present study examines the relationship between persistent MUPS in general practice on the one hand and quality of life, social conditions, and coping on the other hand. Additionally, it is examined how patients with persistent MUPS evaluate the quality of GP-care.

**Methods:**

Data were used from a representative survey of morbidity in Dutch general practice, in which data from the electronic medical records were extracted. A random sample of patients participated in an extensive health interview and completed self-reported measures on social isolation, coping and the quality of GP-care. Patients with persistent MUPS (N = 192) were compared with general practice patients not meeting the criteria for persistent MUPS (N = 7.314), and with a group of patients that visited the GP in comparable rates for medical diagnoses (N = 2.265). Multiple logistic regression analyses were used to control for relevant socio-demographic variables and chronic diseases.

**Results:**

After adjustment for demographics and chronic diseases, patients with persistent MUPS reported more psychological distress, more functional impairment, more social isolation, and they evaluated the quality of GP-care less positive than the other two patient groups. Although the majority of MUPS patients were positive about the quality of GP-care, they more often felt that they were not taken seriously or not involved in treatment decisions, and more often reported that the GP did not take sufficient time. The three groups did not differ with respect to the statement that the GP unnecessarily explains physical problems as psychological ones.

**Conclusion:**

Strengthening MUPS patients' social network and encouraging social activities may be a meaningful intervention in which the GP may play a stimulating role. To further improve MUPS patients' satisfaction with GP-care, GPs may pay extra attention to taking sufficient time when treating MUPS patients, taking the problems seriously, and involving them in treatment decisions.

## Background

Physical symptoms for which no relevant organic pathology can be found after medical evaluation are common in general practice as well as in the general population [1-4]. Such medically unexplained physical symptoms (MUPS) are a burden for the patient because they are associated with increased functional impairments, impaired quality of life, and psychopathology, such as anxiety and depression [3,5,6]. MUPS also burden health care providers through increased health care utilization, and society at large with high costs due to sickness absence and service use [e.g. [7-10]]. Thus, effective management of medically unexplained symptoms is an important public health issue.

Medically unexplained physical symptoms become especially problematic when they become recurrent or chronic. A recent study on persistent MUPS in general practice demonstrated that 2.5% of patients who visited their general practitioner (GP) were regarded as having persistent MUPS, defined as at least four consultations in one year with MUPS [11]. That study focused on socio-demographic characteristics of these patients and their health care utilization, showing that patients with persistent MUPS were older, more often female, from a lower socio-economic status and of non-Western origin, and they consulted the GP more frequently than patients without persistent MUPS or patients who visited the GP with a medical diagnosis [11]. The present study also deals with these patients with persistent MUPS, and is focusing on two topics: a) factors that may be related to persistent MUPS, and b) the way MUPS patients evaluate the quality of GP-care.

The first topic deals with factors that may be associated with the development or persistence of MUPS. Identifying such factors will be helpful for either the development of appropriate and effective interventions or for early identification of persons at risk. Prior research has shown that patient personality, a history of trauma or abuse, and certain demographic characteristics are associated with MUPS [3,7,11-13]. Several theories and a substantial amount of research have identified social support as a factor that directly or indirectly affects health [14,15]. It was, for instance, found that lack of social support was an important risk factor for the persistence of chronic fatigue [16]. Lack of social support has been associated with increased morbidity and frequent attendance in general practice as well [17]. Therefore, low social support may be associated with persistent MUPS in general practice. Furthermore, coping may also be related to the development or continuation of MUPS. Maybe patients with persistent MUPS are more inclined to use specific coping strategies, which may be less adaptive. For instance, research on patients with chronic fatigue syndrome has shown that these patients used more avoidance coping strategies than non-ill controls [18,19]. Until now, however, little quantitative research is available on the relationship between persistent MUPS as presented in general practice and coping or social support.

The second topic focuses on the quality of care as evaluated by MUPS patients. Persistent MUPS may affect the way patients evaluate the quality of care. Several prior studies have demonstrated a strained and problematic relationship between doctors and patients with MUPS. On the one hand, patients with MUPS often feel misunderstood, disbelieved, or rejected by their physician [20,21]. On the other hand, physicians often perceive patients with MUPS as difficult, frustrating, and demanding [e.g. [22-27]]. In such circumstances it can be hypothesized that there is an increased chance of misunderstanding, which may be reflected in less patient satisfaction, a more negative appraisal of the quality of care, or in an increased distrust in health care [28]. It would be helpful to know how patients with persistent MUPS feel about the GP-care they receive. In this way, possible sources of dissatisfaction may be identified, which may help GPs to adjust the care more to patients' needs. Until now, relatively little is known about patient satisfaction and the evaluation of the quality of GP-care among patients with prolonged MUPS compared with other patient groups. Research among general medical patients with somatic symptoms – not necessarily unexplained symptoms – showed that patients with persistent symptoms were more likely to report dissatisfaction [29]. Two other studies on difficult patients, amongst which patients with MUPS, observed that patients who were classified by their doctors as difficult, were less satisfied with their medical care than their non-difficult counterparts [25,26]. Therefore, it is expected that patients with persistent medically unexplained symptoms will be less satisfied with the care they receive from the GP.

The present study focused on persistent medically unexplained symptoms in general practice and used both data from medical records and a survey. The first aim of the present study is to examine relationships between persistent MUPS on the one hand and psychological distress, quality of life, social conditions, and coping on the other hand. The second aim is to investigate the degree of trust MUPS patients have in health care and how they evaluate the quality of GP-care. Patients with persistent MUPS are compared with the other general practice patients, and with a group of patients that visited the GP in comparable rates but for health problems with a medical diagnosis.

## Methods

### Design and procedures

The present study used data collected within the framework of the second Dutch National Survey in General Practice (DNSGP-2), which included a nationwide, representative sample of 195 general practitioners with approximately 400.000 enlisted patients, who were a good representation of the Dutch population in terms of age, gender and type of health insurance [30]. During a period of 12 months, data on all consultations with patients were extracted from the electronic medical records. The GPs recorded all diagnoses/symptoms of their patients, prescribed medications and referrals. Morbidity presented to the GP was classified according to the International Classification of Primary Care (ICPC) and was clustered into episodes of illness [31]. As part of the DNSGP-2, a random sample of 12.699 patients participated in an extensive health interview survey (response rate 64.5%). Questionnaires were administered by trained interviewers during a face-to-face interview. Privacy of the participating persons was guaranteed and in accordance with Dutch legislation, and the study was approved by the Dutch Data Protection Authority. Patients were informed about the study prior to data collection by announcements in the general practice and a personal letter from their GP [30].

### Definition of medically unexplained physical symptoms

In the present study the definition of MUPS consisted of two parts: the content (which ICPC codes are good indicators for MUPS) and the persistent character of such symptoms. We followed the same procedure as in a previous study [11], and we used the study of Robbins and colleagues [32] as a starting point. Robbins et al explored 23 symptoms often associated with functional syndromes. These symptoms are clustered into five syndromes: pain, fatigue, irritable bowel, somatic symptoms of anxiety, and somatic symptoms of depression. Of these 23 symptoms 20 could be classified within the ICPC [11]. An episode of illness was considered as medically unexplained if the episode consisted of one or more of these 20 symptoms, while during the whole year no medical diagnosis (i.e. ICPC-code > 70) had been registered in the relevant episode. Furthermore, to define the persistent character, the patient should have had at least four consultations with medically unexplained symptoms from one of the five above mentioned syndromes. Thus, patients with persistent medically unexplained physical symptoms had at least four consultations with symptoms from one cluster of the 'Robbins-list', without having a consultation for a medical diagnosis within the relevant episode.

### Participants

Of the 12.699 persons who completed the health interview, 9.685 were adults (i.e. 18 years and older), and for 7.506 adults data on episodes of illness were available (78%). Of these, 192 (2.6%) met the criteria for persistent medically unexplained symptoms during the one-year period ('MUPS group'). The MUPS patients who participated in the health interview were representative of the whole group of MUPS patients that took part in the DNSGP-2 in terms of age, gender, and type of health insurance. These MUPS patients are compared with the remaining patients that did not meet the criteria for MUPS ('non-MUPS group'; N = 7.314). Because the MUPS group was selected on the basis of their relatively frequent use of GP-care (i.e. at least four consultations), a second reference group was constructed out of the 7.314 non-MUPS patients, which consisted of patients who did not meet the MUPS criteria but had at least four contacts with their GP for medical diagnoses (with the exception of psychological diagnoses). This so-called 'Diagnosis group' consisted of 2.265 patients.

A more detailed description of the clinical characteristics of the MUPS group can be found in a previous study [11], which made use of the same definition of persistent MUPS and GP-data. The study showed that MUPS patients had significantly more contacts with their GP than both the Diagnosis and non-MUPS group (15.9, 10.9 and 5.1 respectively). Additionally, they experienced significantly more episodes of illness (7.6, 6.1 and 3.5 respectively) and more episodes of illness which the GP labeled as psychological than both other patient groups (0.62, 0.26 and 0.19 respectively) [11].

### Measures

The questionnaire included the following socio-demographic characteristics and lifestyle variables: age, gender, marital status (unmarried/married/divorced/widowed), type of health insurance (private/public), immigrant status (native/non-native), educational level (low/middle/high), smoking (never/ever/current), heavy drinking (at least once a week more than five glasses of alcohol [33]), obesity (Body Mass Index of 30 and higher [34]), and insufficient physical activity (less than 5 days a week at least 30 minutes of moderately intensive exercise activities, such as cycling, walking, or gardening [33]).

To measure current health-related quality of life, a Dutch translation of the Short Form Health Survey 36 (SF-36) was used [35,36]. The SF-36 measures the following aspects of quality of life: physical functioning (10 items), role limitations due to physical health problems (4 items), bodily pain (2 items), general health (5 items), vitality (4 items), social functioning (2 items), role limitations due to emotional problems (3 items), and general mental health (5 items). According to the guidelines, the raw scale scores were transformed to a 0 to 100 scale, with higher scores indicating higher levels of functioning or well-being.

The Dutch 12-item version of the General Health Questionnaire (GHQ-12) was included to screen for symptoms of common mental disorder [37,38]. A cut-off score of 2 or higher is considered as an increased risk of psychopathology.

The health interview included questions on the presence of chronic conditions, such as asthma/COPD, high blood pressure, rheumatoid arthritis, myocardial infarction, vascular disorder, and diabetes [30]. Answer categories were 'yes' or 'no', and 12 different chronic conditions were investigated for the present study. A summary score counted the number of self-reported chronic diseases.

Coping style was measured using the Dutch adjustment of the coping inventory for stressful situations (CISS-21) [39,40]. The CISS-21 consists of three scales: task oriented coping (7 items), emotion oriented coping (7 items), and avoidance oriented coping (7 items). A five point Likert scale (1 = not at all; 5 = very much) was used. Mean scores were calculated, and a higher score indicates a greater use of the coping style.

The degree of social isolation was measured using six items that were based on the UCLA Loneliness Scale, e.g. 'I feel part of a group of friends', 'I feel isolated from others', 'There are people who really understand me' [41]. A summary score counted the degree of social isolation (possible score range 6–18), with a higher score representing a higher degree of social isolation.

To assess how patients evaluated the quality of care from their GP, a 22-item scale (Quote) was used [42]. Two subscales can be derived concerning: 1) the content of the GP-care consisting of 12 items (e.g. 'the GP always takes me seriously'; 'the GP provides clear information regarding the treatment'; 'the GP takes sufficient time to talk'), and 2) the structure and organization of the GP-care consisting of 10 items (e.g., 'a waiting time of less than 15 minutes', 'good privacy in the practice'). Mean scores were calculated and could range from 1 (the GP-care falls completely short of expectations) to 4 (the GP-care meets all expectations).

To explore the degree of trust in health care in general, patients were asked to give a number between 1 and 10 for their trust in current health care, future health care, and current medical possibilities. A '1' represented no trust at all, whereas a '10' represented a lot of trust. Additionally, three items investigated the degree of trust in general practitioners, medical specialist, and hospitals. Patients could answer on a four point scale, ranging from 1 (very little trust) to 4 (a lot of trust).

### Data analyses

Chi-square tests and t-tests were used to compare the MUPS group with the non-MUPS and diagnosis group with respect to demographic characteristics, lifestyle, chronic diseases, functional health status, psychological distress, coping strategies, social isolation, trust in health care and their evaluation of the quality of health care. Multiple logistic regression analyses were then performed to control for relevant socio-demographic characteristics and chronic diseases.

## Results

With respect to demographic characteristics, MUPS patients differed especially when compared with the non-MUPS group. MUPS patients were more often female, were significantly older, more of them had public health insurance, and they were more often widowed than patients from the non-MUPS group (see Table 1). Compared with both non-MUPS patients and patients with a medical diagnosis, MUPS patients had a lower educational level.

**Table 1:** Socio-demographic characteristics and lifestyle.

	MUPS group			Diagnosis group			Non-MUPS group			p
Socio-demographics	N	n	%	N	n	%	N	n	%	
Male gender	192	64	33.3	2265	858	37.9	7314	3026	41.4	p = .24^a^; p < .05^b^
Mean age (SD)	192	56.3	(17.81)	2265	57.2	(17.2)	7314	49.7	(17.14)	p = .49^a^; p < .001^b^
Public health insurance	192	148	77.1	2264	1623	71.7	7308	4958	67.8	p = .13^a^; p < .01^b^
Marital status	192			2263			7308			p = .25^a^; p < .001^b^
Unmarried		28	14.6		284	12.5		1417	19.4	
Married/living together		115	59.9		1515	66.9		4889	66.9	
Divorced		15	7.8		135	6.0		385	5.3	
Widowed		34	17.7		329	14.5		617	8.4	
Immigrant status	170			2041			6518			p = .87^a^; p = .95^b^
Native		153	90.0		1851	90.7		5896	90.5	
Non-native		17	10.0		190	9.3		622	9.5	
Educational level	192			2261			7304			p < .01^a^; p < .001^b^
Low		64	33.3		533	23.6		1154	15.8	
Medium		111	57.8		1395	61.7		4674	64.0	
HBO/academic		17	8.9		333	14.7		1476	20.2	
Life style										
Heavy drinking	192	14	7.3	2265	157	6.9	7314	762	10.4	p = .97^a^; p = .20^b^
Smoking	191			2262			7306			p = .39^a^; p = .22^b^
Never		55	28.8		757	33.5		2415	33.1	
Ever		82	42.9		934	41.3		2694	36.9	
Currently		54	28.3		571	25.2		2197	30.1	
Insufficient exercise	190	93	48.9	2255	1047	46.4	7295	3069	42.1	p = .55^a^; p = .07^b^
Obesity	192	32	16.7	2265	344	15.2	7314	832	11.4	p = .66^a^; p < .05^b^

^a ^t-test/chi-square test MUPS versus Diagnosis group.

^b ^t-test/chi-square test MUPS versus non-MUPS group.

Hardly any differences were found between the three patient groups with respect to lifestyle habits. One exception was found: MUPS patients more frequently had obesity (16.7%) when compared with the non-MUPS group (11.4%). However, after controlling for demographic characteristics, obesity no longer differed significantly between MUPS and non-MUPS patients (OR = 1.30; 95% confidence interval = 0.87–1.93).

MUPS patients reported increased levels of psychological distress and more functional impairments in different aspects of their lives when compared with the two other reference groups (see Table 2). Forty-two percent of MUPS patients scored above the GHQ cut-off score, while 24% of the non-MUPS group did so (χ^2 ^= 31.1; p < .001). Also compared with patients with a medical diagnosis (29%), more MUPS patients were potential cases according to the GHQ (χ^2 ^= 14.0; p < .001).

**Table 2:** Perceived health status, health-related quality of life, coping strategies, and loneliness.

	MUPS group			Diagnosis group			Non-MUPS group			p
	N	M	SD	N	M	SD	N	M	SD	
Psychological distress (GHQ-12)^a^	191	10.92	5.04	2261	9.58	4.68	7291	9.10	4.47	p < .001^c^; p < .001^d^
Health related quality of Life (SF-36)^b^										
Physical functioning	192	67.8	30.1	2257	75.4	27.0	7295	84.6	22.6	p < .001^c^; p < .001^d^
Social functioning	192	83.3	31.3	2260	90.2	28.6	7301	95.7	25.0	p < .01^c^; p < .001^d^
Role limitations physical	192	56.1	44.1	2261	68.5	40.8	7301	77.7	36.6	p < .001^c^; p < .001^d^
Role limitations emotional	192	75.9	39.5	2261	86.5	30.2	7302	89.0	27.4	p < .001^c^; p < .001^d^
Mental functioning	192	69.1	20.7	2259	76.7	17.3	7300	79.4	16.2	p < .001^c^; p < .001^d^
Vitality	192	56.3	22.8	2257	64.5	20.3	7290	68.5	19.1	p < .001^c^; p < .001^d^
Pain	192	62.7	29.7	2264	73.0	26.7	7307	79.6	23.9	p < .001^c^; p < .001^d^
General health	192	53.8	22.4	2259	59.8	21.2	7291	67.5	20.3	p < .001^c^; p < .001^d^
Chronic diseases	192	1.18	1.19	2265	1.27	1.22	7314	0.77	1.04	p = .39^c^; p < .001^d^
Coping style (CISS-21)^e^										
Task oriented	188	3.41	0.83	2229	3.46	0.79	7233	3.55	0.76	p = .44^c^; p < .05^d^
Emotion oriented	188	2.49	0.86	2226	2.40	0.81	7233	2.39	0.80	p = .12^c^; p = .08^d^
Avoidance oriented	189	2.42	0.89	2234	2.34	0.91	7247	2.42	0.91	p = .24^c^; p = .98^d^
Social isolation/loneliness (UCLA)^f^	190	8.26	2.58	2253	7.81	2.12	7292	7.57	2.07	p < .05^c^; p < .001^d^

^a ^GHQ scores can range from 0 to 36 (according to 0123 coding). ^b ^SF-36 scores can range between 0–100. ^c ^T-test MUPS versus Diagnosis group.^d ^T-test MUPS versus non-MUPS group. ^e ^Scores on the coping scale can range between 1–5. ^f ^Scores on the social isolation scale can range between 6–18.

In comparison with the non-MUPS patients, MUPS patients were more likely to report at least one chronic disease (62.5% versus 47.4%; χ^2 ^= 16.5; p < .001). However, after controlling for demographic characteristics, this difference was no longer statistically significant (OR = 1.39; 95% confidence interval = 1.00–1.92). MUPS patients did not differ significantly from the diagnosis group (68.8%; χ^2 ^= 2.9; p > .05) with respect to chronic diseases.

With respect to coping style, only a small significant difference was found between the MUPS patients and patients from the non-MUPS group (see Table 2). MUPS patients were less inclined to use task oriented coping than the non-MUPS patients. Furthermore, MUPS patients reported more social isolation than the other two patient groups.

The three patient groups did not differ very much regarding their trust in health care, medical possibilities, and trust in specified health care providers (see Table 3). The only difference observed was that slightly more MUPS patients reported distrust in medical specialists when compared with the other groups. In comparison with the other patients, MUPS patients were less positive about the quality of GP-care. This difference was only observed for content-related aspects of GP-care.

**Table 3:** Respondents' opinions regarding their trust in health care and the quality of GP-care.

	MUPS group			Diagnosis group			Non-MUPS group			p
(Very) little trust in health care providers	N	n	%	N	n	%	N	n	%	
General practitioners	190	35	18.4	2242	303	13.5	7246	1033	14.3	p = .08^a^; p = .13^b^
Medical specialists	189	32	16.9	2200	260	11.8	7105	819	11.5	p = .05^a^; p < .05^b^
Hospitals	189	37	19.6	2201	357	16.2	7141	1222	17.1	p = .28^a^; p = .43^b^
Trust in health care^c^		M	SD		M	SD		M	SD	
Current health care	189	6.46	1.83	2227	6.62	1.73	7230	6.55	1.70	p = .22^a^; p = .50^b^
Future health care	186	6.10	2.09	2195	6.19	1.76	7160	6.18	1.74	p = .59^a^; p = .61^b^
Current medical possibilities	184	7.52	1.37	2217	7.68	1.26	7212	7.69	1.22	p = .09^a^; p = .06^b^
Quality of GP-care^d^										
Quote – Content	189	3.32	0.53	2215	3.44	0.45	7098	3.43	0.44	p < .01^a^; p < .01^b^
Quote – Organisation	187	3.15	0.47	2215	3.16	0.43	7056	3.14	0.43	p = .70^a^; p = .87^b^
Quote- Total	189	3.24	0.44	2222	3.32	0.39	7095	3.30	0.38	p < .05^a^; p = .08^b^

^a ^t-test/chi-square MUPS versus Diagnosis group.^b ^t-test/chi-square MUPS versus non-MUPS group. ^c ^Scores for trust in health care can range between 1–10. ^d ^Scores for quality of GP-care can range between 1–4.

The multivariate analyses showed that, after adjustment for demographic characteristics and chronic diseases, MUPS patients still reported more psychological distress, more functional impairments, they reported more social isolation, and evaluated the quality of received GP-care as less favorable than the other two groups (see Table 4). After adjusting for demographics and chronic diseases, MUPS patients did no longer differ significantly from the non-MUPS group with respect to task oriented coping style.

**Table 4:** Multiple logistic regression analyses to identify characteristics of MUPS versus Diagnosis and non-MUPS group.

	MUPS vs Diagnosis	MUPS vs non-MUPS
	Adjusted OR^a ^(95% CI)	Adjusted OR^a ^(95% CI)
Health-related quality of life (SF-36)		
Physical functioning	1.56 (1.10–2.21)	2.16 (1.52–3.06)
Social functioning	1.66 (1.18–2.33)	2.22 (1.59–3.10)
Role limitations physical	1.65 (1.21–2.25)	2.36 (1.74–3.19)
Role limitations emotional	2.06 (1.44–2.94)	2.58 (1.83–3.65)
Mental functioning	2.21 (1.57–3.11)	2.66 (1.91–3.70)
Vitality	2.05 (1.49–2.83)	2.84 (2.08–3.88)
Pain	2.02 (1.48–2.76)	2.94 (2.15–4.00)
General health	1.78 (1.30–2.44)	2.57 (1.88–3.52)
Psychological distress (GHQ-12)	1.68 (1.23–2.28)	2.20 (1.63–2.97)
Coping style (CISS-21)		
Task oriented	0.94 (0.77–1.16)	0.91 (0.75–1.12)
Social isolation	1.09 (1.02–1.16)	1.08 (1.02–1.15)
Quality of care		
Quote content	0.55 (0.38–0.80)	0.60 (0.42–0.86)

^a ^Odds ratios adjusted for gender, age, educational level, health insurance type, marital status (married/living together versus unmarried/divorced/widowed), and chronic diseases (yes/no).

To gain more insight into the aspects of GP-care that MUPS patients evaluated less positively, we examined the items that constitute the content-related quality of care scale (see Table 5). MUPS patients more often felt that the GP didn't take them seriously, they more often were not involved in decisions regarding the treatment of their complaints, they felt that their GP was less prepared to talk about all their problems or about matters that had gone wrong, and they more often felt that their GP did not take sufficient time. Additionally, compared with the other patient groups, MUPS patients more often reported that the GP didn't refer them immediately to medical specialists.

**Table 5:** Quality of GP-care: dissatisfaction with content-related aspects of GP-care.

	MUPS group	Diagnosis group	Non-MUPS group	p
The GP:	%	%	%	
does not always takes me seriously	15.8	9.3	9.1	p < .01^a^; p < .01^b^
does not always involve me in the decision regarding treatment	13.4	7.9	7.1	p < .05^a^; p < .01^b^
does not always provide a clear explanation regarding prescribed medications	10.6	9.9	9.3	p = .85^a^; p = .61^b^
does not always clearly explain to me what's the matter	9.0	6.1	5.3	p = .14^a^; p < .05^b^
is not always prepared to talk about all my problems	25.7	13.8	14.7	p < .001^a^; p < .001^b^
explains physical problems unnecessarily as psychological ones	28.7	24.5	23.4	p = .24^a^; p = .12^b^
is not always prepared to visit at home	14.2	12.0	13.3	p = .43^a^; p = .80^b^
does not always take sufficient time to talk	17.5	10.6	10.8	p < .01^a^; p < .01^b^
does not always clearly explain the purpose and nature of treatment	4.8	5.1	4.9	p = 1.0^a^; p = 1.0^b^
is not always prepared to talk about mistakes or matters that have gone wrong	14.7	10.0	9.6	p = .06^a^; p < .05^b^
does not always clearly explain the results of an examination	6.0	5.3	5.2	p = .82^a^; p = .75^b^
does not always give me access to my medical record when I request this	10.0	7.5	6.6	p = .26^a^; p = .09^b^
does not always refer me immediately to medical specialists when I request this	23.7	16.4	17.2	p < .05^a^; p < .05^b^

^a ^Chi-square test MUPS versus Diagnosis group.

^b ^Chi-square test MUPS versus non-MUPS group.

## Discussion

Patients who consult their GP for persistent medically unexplained symptoms differ significantly from other general practice patients and from patients who consult the GP in comparable rates but for medical diagnoses. After adjustment for demographic characteristics and chronic diseases, MUPS patients reported significantly more distress and impaired quality of life, they felt socially more isolated, and were less positive about the quality of GP-care. No significant differences were found with respect to lifestyle, coping style and trust in health care.

Consistent with prior studies, patients with persistent MUPS reported increased psychological problems and more functional impairments in different aspects of their lives when compared with other general practice patients [3,6]. The worse health status reported by MUPS patients was not affected by differences in unhealthy habits, such as decreased levels of physical activity, increased levels of alcohol consumption or smoking behavior, since MUPS patients did not differ from the other patients with respect to lifestyle. The definition of sufficient physical activity used in the present study is based on Dutch guidelines and refers to 30 minutes of moderately intensive exercise for at least five days a week [43]. It may be possible that using a less stringent definition of insufficient physical activity or using a definition referring to intensive physical exercise would have resulted in differences between groups.

It has been estimated that at least 40% of patients with MUPS have psychiatric problems [44]. In line with this, the present study found that about 40% of patients with persistent medically unexplained symptoms scored above the GHQ cut-off score, indicating that they might have a psychological disorder. This percentage was significantly higher even when compared with patients visiting the GP in comparable rates but for medical diagnoses. It should be remembered, however, that there is no one-to-one relationship between MUPS and psychopathology, and many patients with MUPS have no definite psychological illness [45].

Contrary to expectations, no significant differences were found with respect to coping style between MUPS patients and the other two patient groups, suggesting that coping style as measured in the present study does not play an important role with respect to persistent medically unexplained symptoms. Prior research in primary care on chronic fatigue syndrome, which also concerns medically unexplained symptoms, demonstrated that patients with chronic fatigue syndrome were more inclined to use avoidance coping strategies [19]. A possible explanation for the lack of an association between coping and persistent MUPS in the present study may be the way coping was operationalized. It may be more appropriate to measure coping in relation to health or symptoms instead of measuring coping strategies in general.

In line with expectations, patients with persistent MUPS fared worse with respect to their social lives; they felt more alone and socially isolated than non-MUPS patients. Even though MUPS patients and patients with medical diagnoses may both experience limitations in social and societal participation due to their health problems, we observed that MUPS patients felt lonelier than patients with a medical diagnosis. To our knowledge, little is known about the relationship between persistent MUPS as presented in general practice and social support. Research on frequent attenders in general practice demonstrated that frequent attendance was associated with social factors, such as low social support or unemployment [17]. However, the results are not unambiguous [17,46]. The findings of the present study suggest that strengthening MUPS patients' social networks and encouraging social activities can be a relevant aspect of interventions.

The vast majority of MUPS patients were positive about the quality of GP-care. We did find, however, that MUPS patients were less positive about the GP-care than the other two patient groups. This is in line with a prior study on difficult patients (amongst which patients with MUPS), which showed that although difficult patients were less satisfied than other patients, only 10 percent were markedly dissatisfied with the care they received [25]. Contrary to the positive evaluation of GP-care, a study conducted in specialist care showed that two-thirds of patients with chronic fatigue syndrome were dissatisfied with the quality of medical care received [47]. Patient satisfaction may differ between health-care settings. For instance, especially the most severe or complex symptomatology, or patients who are less satisfied with primary care may find their way to specialist care. Therefore, patient dissatisfaction may be more prevalent in specialist care than in primary care. In the present study, the less positive evaluation referred to content-related aspects of GP-care and confirmed the idea that patients with MUPS more often feel that they are not being properly heard or taken seriously. These differences may reflect specific needs of MUPS patients; they may reflect the fact that GPs frequently find MUPS patients difficult and that GPs might have less patience with these patients; or they may reflect doctor-patient conflicts, frequently reported with MUPS patients [e.g. [21,24,27]].

Strikingly, while prior work has frequently suggested that many MUPS patients feel misunderstood because the GP labels their symptoms as psychological rather than physical [6,46,48], the present study negates this idea. The same proportion (about a quarter) of MUPS patients, non-MUPS patients, and patients with medical diagnoses reported that the GP explained physical problems unnecessarily as psychological ones.

The present study has some methodological limitations. A first concern is the representativeness of the study sample. Only a small patient sample was invited to participate in the health survey. Although the response rate was relatively high (64%), it is possible that selection has occurred, which may limit the generalisability of the results. Secondly, the study design is cross-sectional, therefore, we cannot make causal inferences about the relationships observed. Longitudinal cohorts are required to further unravel the relationships between persistent MUPS and the other variables.

Thirdly, the definition of persistent MUPS as used in the present study has not been validated. The MUPS patients of the present study did, however, resemble MUPS patients of prior studies with respect to increased psychological distress and functional impairments [3,5,6]. Additionally, the patients with persistent MUPS were also characterized by an increase in health care utilization and more episodes of illness (for medical as well as psychological problems) [11]. The criterion of at least four consultations with medically unexplained symptoms in one year is rather arbitrary. Until now, there is no unambiguous definition of MUPS, which makes comparisons difficult. Additionally, the concept of MUPS is complex because different factors may influence whether or not a physician views symptoms as 'medically unexplained', such as doctor-patient communication, patient characteristics, GPs' perception of the interaction with the patients, or the way the symptoms are presented [49]. However, the approach in the present study has some advantages. Firstly, the longitudinal data collected in the course of one year made it possible to focus on long-term lack of a medical explanation. Additionally, the GP was not asked to label each patient's symptoms as either MUPS or no MUPS. This approach may be less susceptible for GP-bias.

The present study also has important strengths. First of all, the study included a nationwide sample of patients in general practice, and could compare MUPS patients with two different reference groups. Furthermore, until now, little is known about the relationships between *persistent *MUPS in general practice on the one hand, and coping, social support, trust in health care, and the quality of GP-care on the other hand. Therefore, the present study adds valuable knowledge on patients with persistent medically unexplained symptoms in general practice.

## Conclusion

Medically unexplained physical symptoms are common in general practice, and although persistent MUPS appear to be less prevalent in general practice, they are associated with increased rates of GP consultation. Therefore, dealing with MUPS is an important part of the GP's work. Most patients with persistent MUPS in general practice were positive about the quality of GP-care. Some additional profit can be gained if GPs make sure to take sufficient time when treating MUPS patients, to take the patients' problems seriously and to involve MUPS patients in treatment decisions. This may help to further improve MUPS patients' satisfaction with the care they receive. Since patients with persistent MUPS feel socially more isolated than the other general practice patients, encouraging social activities may be a meaningful intervention. In this respect the GP may play a stimulating role as well. Additional longitudinal research is necessary to further unravel the relationships between persistent MUPS and both individual factors (e.g. coping, illness perceptions), environmental factors (e.g. social support, loneliness), and the way MUPS patients feel about the GP-care they receive.

## Competing interests

The author(s) declare that they have no competing interests.

## Authors' contributions

AD was responsible for the data analysis and interpretation of the data, and wrote the article. PV made substantial contributions to the conception and design of the study, and critically reviewed the manuscript. All authors have read and approved the final manuscript.

## Pre-publication history

The pre-publication history for this paper can be accessed here:


